# Intravoxel incoherent motion and ADC measurements for differentiating benign from malignant thyroid nodules: utilizing the most repeatable region of interest delineation at 3.0 T

**DOI:** 10.1186/s40644-020-0289-2

**Published:** 2020-01-22

**Authors:** Minghui Song, Yunlong Yue, Yanfang Jin, Jinsong Guo, Lili Zuo, Hong Peng, Queenie Chan

**Affiliations:** 1 Department of MR, Beijing Shijitan Hospital, Capital Medical University, Peking University Ninth School of Clinical Medicine, Tieyilu #10, Haidian District, Beijing, 100038 China; 2 Department of Otolaryngology, Beijing Shijitan Hospital, Capital Medical University, Peking University Ninth School of Clinical Medicine, Beijing, China; 3 Philips Healthcare, Shatin, New Territories Hong Kong, China

**Keywords:** Diffusion magnetic resonance imaging, Thyroid nodule, Reproducibility of results

## Abstract

**Background:**

There is a growing need for a reproducible and effective imaging method for the quantitative differentiation of benign from malignant thyroid nodules. This study aimed to investigate the performances of intravoxel incoherent motion (IVIM) parameters and the apparent diffusion coefficient (ADC) in differentiating malignant from benign thyroid nodules derived from the most repeatable region of interest (ROI) delineation.

**Methods:**

Forty-three patients with 46 pathologically confirmed thyroid nodules underwent diffusion-weighted imaging (DWI) with 8 *b* values. Two observers measured the intravoxel incoherent motion (IVIM) parameters (*D*, *f* and D*) and the apparent diffusion coefficient (ADC), ADC_600_ and ADC_990_ values using whole-lesion (W-L) ROI and IVIM parameters using single-section (S-S) ROI delineation. The intraclass correlation coefficients (ICCs) and Bland-Altman plots were used to evaluate the intra- and interobserver variability. The diagnostic performance of these parameters was evaluated by generating receiver operating characteristic (ROC) curves.

**Results:**

The ICC values of all IVIM with W-L ROI delineation were higher than those with S-S ROI delineation, and excellent intra- and interobserver reproducibility was obtained. According to the Bland-Altman plots, the 95% limits of agreement of the IVIM parameters determined by the W-L ROIs revealed smaller absolute intra- and interobserver variability than those determined by S-S ROIs. The *D* and ADC_600_ values obtained from the W-L ROIs were the most powerful parameters in differentiating benign from the malignant nodules [area under the ROC curve = 0.962 and 0.970, *P* = 0.771].

**Conclusions:**

The W-L ROI of the thyroid was considered an effective method for obtaining IVIM measurements with excellent reproducibility for differentiating benign from malignant nodules.

## Background

Thyroid cancer is the most common malignant disease of the endocrine system, and its incidence is rapidly increasing [1]. Although most thyroid cancers are well differentiated papillary carcinomas [2], due to the heavy psychological and mental burden of this disease, most patients tend to undergo surgery. Because the thyroid gland is a superficial organ, the application of high-resolution ultrasonography (US) in thyroid diseases has obvious advantages. However, thyroid nodules evaluated by US or US-guided fine needle aspiration biopsy are reportedly operator dependent, and no single sonographic feature has sufficient diagnostic value. According to a systematic review and meta-analysis of observational studies, the accuracy of US in predicting malignant thyroid nodules has a sensitivity in the range of 26~87% and a specificity in the range of 40~93% [3].

Diffusion-weighted imaging (DWI) is a functional magnetic resonance imaging (MRI) modality. The apparent diffusion coefficient (ADC) value is an objective parameter of DWI that reflects free water diffusion and provides a quantitative measurement of the motion of water molecules in various diseases. Previous studies have demonstrated the potential of ADC in distinguishing benign from malignant thyroid nodules and other thyroid diseases [4, 5]. On the basis of a recent meta-analysis of studies differentiating benign from malignant thyroid nodules, the sensitivity of quantitative DWI and ADC was 0.90~0.91, and the specificity was 0.93~0.95 [6, 7]. However, the diagnostic threshold demonstrated considerable variability (0.36–2.56 × 10^− 3^ mm^2^/s) among studies. Considering the limitations of applying lower or higher *b* values in a linear form, investigations into head and neck cancers to differentiate benign from malignant neoplasms and predict treatment responses have been performed using intravoxel incoherent motion (IVIM), which could better reflect true diffusion in the tissue [8, 9]. Recently, IVIM applications in thyroid and related diseases have been increasingly reported [10–12]. Texture analyses of thyroid nodules based on magnetic resonance imaging have also been reported [13]. Based on large-sample and multi-centre research, artificial intelligence and machine learning could gradually be applied to magnetic resonance imaging of thyroid nodules.

Accurate image segmentation is the premise of radiomics analyses. Most previous studies adopted a manual single-section (S-S) region of interest (ROI) analysis for the quantitative assessment of thyroid nodules to avoid necrotic, cystic and micro-calcified areas [6, 7, 14–17], while few studies used whole-lesion (W-L) ROI analyses [12, 18–20]. Notably, the appropriate ROI size and positioning have considerable influence on the quantitative parameters of tumour and interobserver variability [21, 22]. Furthermore, some studies used W-L ROI IVIM for rectal and hepatic tumours, believing that this method can accurately reflect and evaluate the information of the lesions [21, 23, 24]. At present, there are no comparative studies on the delineation of thyroid nodules in the literature.

Therefore, this study aimed to compare the reproducibility and repeatability of the IVIM parameters derived from W-L ROI and S-S ROIs and the IVIM and ADC parameters derived from W-L ROIs to distinguish malignant from benign thyroid nodules.

## Methods

### Study design

This study was approved by the local ethics committee. Inclusion criteria were as follows: (1) thyroid nodules or masses detected by physical examination or incidentally; (2) thyroid nodules > 1 cm detected by ultrasonography; (3) clinicians and/or patients believed that the lesion could be removed surgically; and (4) written informed consent for MRI examination was provided. For some nodules that were suspected to be benign, surgical treatment was performed according to the patients’ preferences or symptoms. Patients were excluded if they had any MRI-incompatible metallic devices or motion artefacts during the MRI examination.

From April 2017 to May 2018, this study enrolled 43 patients (6 men, 37 women; mean age, 42 ± 11 years; age range, 18–68 years). A total of 46 thyroid nodules were evaluated. The pathological findings confirmed 24 benign nodules and 22 malignant nodules. Benign nodules: nodular goitre (*n* = 22) and Hurthle cell adenoma (*n* = 2). Malignant nodules: papillary thyroid cancer (*n* = 20), medullary thyroid carcinoma (*n* = 1) and anaplastic carcinoma (*n* = 1). Furthermore, 40 patients had solitary thyroid nodules, while 3 patients had 2 nodules. No significant difference was found between the mean major diameter of the benign nodules and that of the malignant nodules (malignant nodules: 3.06 ± 1.22 cm; benign nodules: 3.18 ± 1.65 cm, *P* > 0.05).

### MRI scan protocol

All patients underwent preoperative examinations (Philips 3.0 T Ingenia, Philips Medical System, The Netherlands). Routine T1-weighted imaging (T1WI), T2-weighted imaging (T2WI) and DWI were acquired using an 8-channel phased-array carotid coil. T1/T2-weighted turbo spin-echo sequences were used to visualize the morphologic features of thyroid nodules (FOV, 22 × 22 cm; voxel size, 0.85 × 0.85 mm/0.76 × 0.76 mm; number of slices, 20; slice thickness, 4 mm; slice spacing, 0.6 mm; repetition time (TR) and echo time (TE) of 525/36 ms and 3600/100 ms; turbo factor, 8 and 22; flip angle, 90°; and total acquisition time, 6:34 min) and to gain sufficient anatomical information. An rFOV DWI examination using a two-dimensional (2D) spatially selective echo-planar radiofrequency (RF) excitation technique was performed with the following parameters: TR/TE, 1351/69 ms; turbo factor, 29; EPI factor, 29; FOV, 160 × 47 mm; voxel size, 1.50 × 1.50 mm; NSA 4; 10 to 15 slices with thicknesses of 5.0 mm and a 1.0-mm gap. Eight *b* values (0, 20, 50, 100, 200, 400, 600, and 990 s/mm^2^) were used, and the acquisition time was 5:37 min.

### Image analysis

Image analysis was performed using MATLAB scripts (The MathWorks, Natick, MA, USA) that provided the D, D*, f, and ADC metrics mapped on a pixel-by-pixel basis. The images were independently analysed by two radiologists (one radiologist with 10 years of experience in MRI radiology and one radiologist with 23 years of experience) who were blinded to the clinical and histopathological data.

**Fig. 1:**
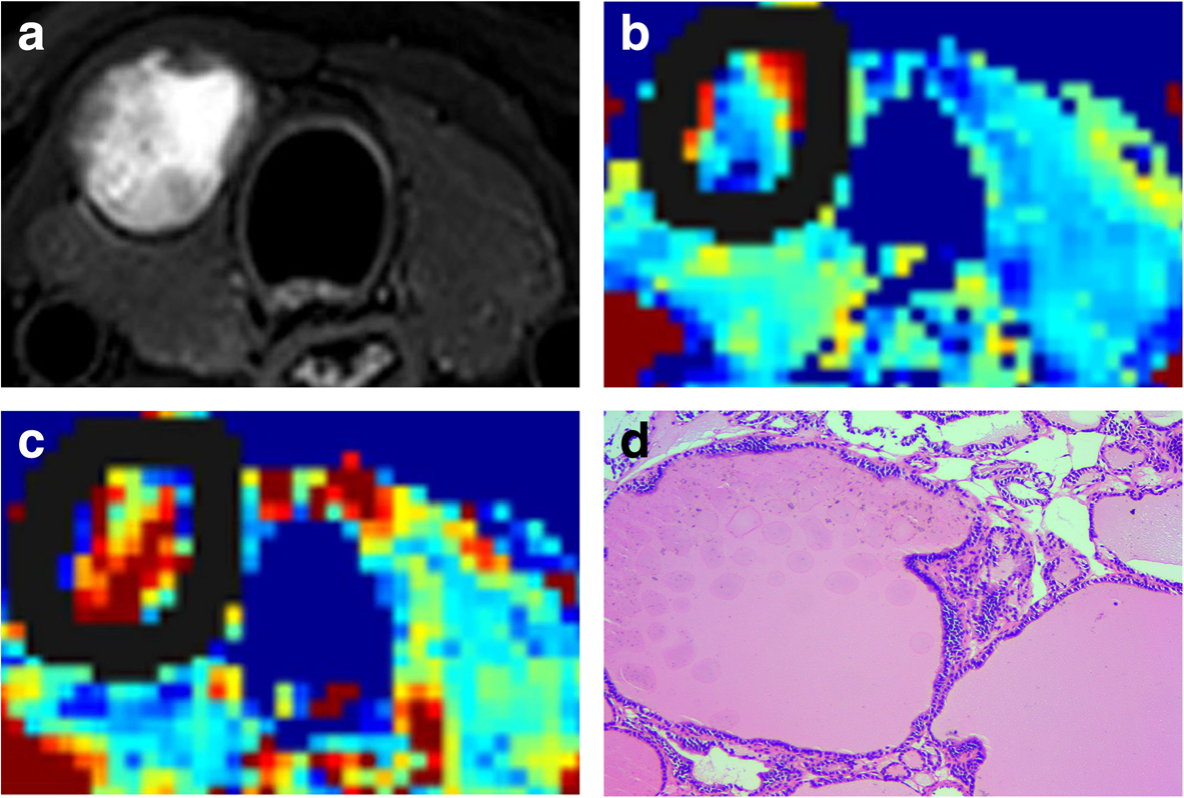
Images of a 36-year-old man with right lobe nodular goitre (central part with cystic necrosis). **a.** Axial T2-weighted with selected partial inversion recovery (SPIR) image. **b-c.** IVIM colour map (*D* and *f*). *D* = 1.28 × 10^− 3^ mm^2^/s, *f* = 39.76%. Solid lines show the nodule border containing cystic necrosis. **d.** Follicles of varying sizes were filled with colloid (HE, × 200)

**Fig. 2:**
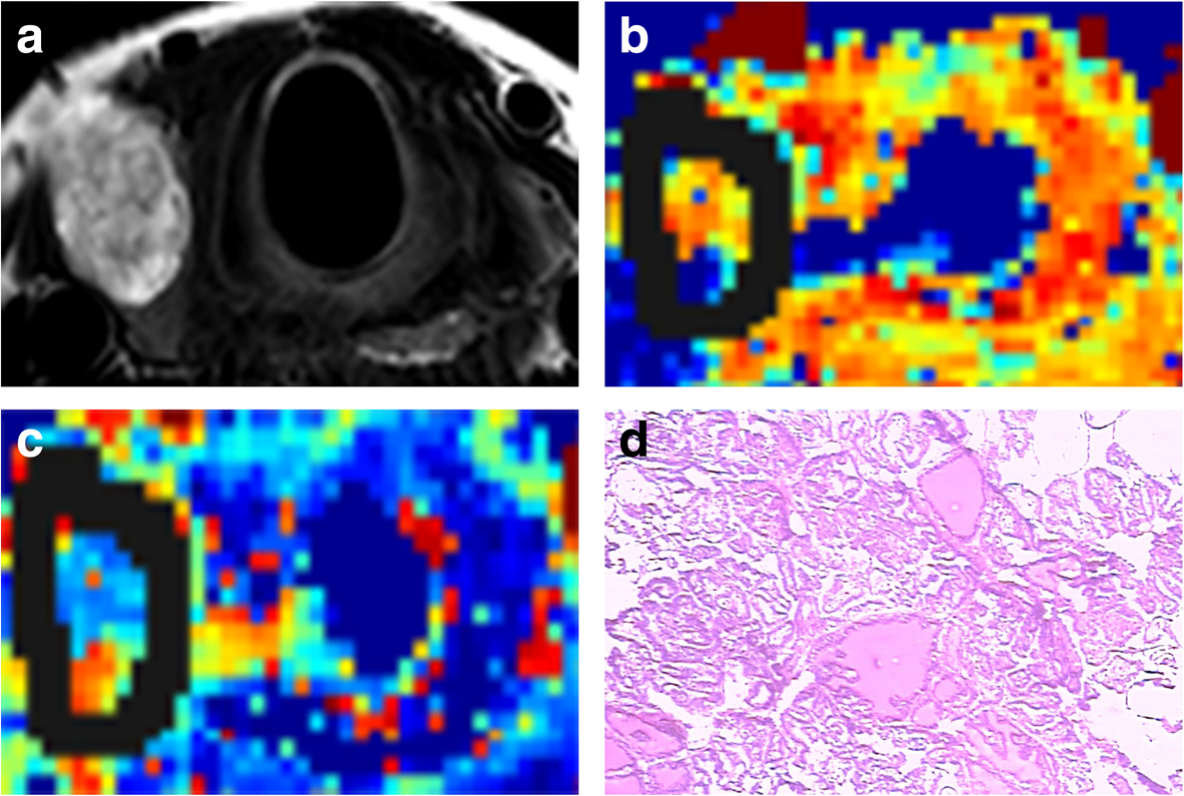
Images of a 65-year-old woman with right lobe papillary thyroid cancer (eccentric part containing cystic necrosis). **a.** Axial T2-weighted image. **b-c.** IVIM colour map (*D* and *f*). *D* = 0.83 × 10^− 3^ mm^2^/s, *f* = 32.15%. Solid lines show borders containing cystic necrosis. **d.** Nuclei arranged closely in papillary thyroid carcinoma (HE, × 100)

This study adopted two methods of delineation: W-L ROI and S-S ROI. For the W-L ROI delineation, a 3D ROI was traced along the margins of the whole-lesion slice by slice, including the cystic, necrotic, and haemorrhagic portions, and obtained at *b* = 0 s/mm^2^, with reference to the corresponding T2WI (Figs. 1 and 2). The W-L ROI delineation trajectory was then saved. For the S-S ROI delineation, the same two radiologists independently drew two sample round/oval-shaped ROIs ranging from 20 to 60 pixels in size within the most solid tumour part (in the *b* = 0 s/mm^2^) of the two independent tumour-containing slices, which were drawn as large as possible while avoiding cystic lesions, necrotic tissues, and vessels. Repeated measurements using the two ROI methods were performed at an interval of 4 weeks by one radiologist.

The ROIs obtained from these two methods were applied in the bi-exponential IVIM model. Diffusivity values were set as follows: 1. *D* (true diffusion, × 10^− 3^ mm^2^/s); 2. D* (pseudo-diffusion, × 10^− 3^ mm^2^/s); and 3. *f* (fractional perfusion related to microcirculation, %). The IVIM model equation was as follows: *Sb*/*S*0 = (1 − *f*) exp. (− *bD*) + exp. (− bD*) [25]. All three parameters were derived by direct curve fitting, and no cut-off values were used. For curve fitting, both *D* and D* were set to be positive and were smaller than 0.01, while D* was larger than *D*. In addition, *f* ranged from 0 to 1.

The mono-exponential ADC was noted as the ADC (× 10^− 3^ mm^2^/s). The ADC was calculated according to the mono-exponential fit of the signal intensity using *b* = 0 s/mm^2^ and 600 s/mm^2^ (ADC_600_) and *b* = 0 s/mm^2^ and 990 s/mm^2^ (ADC_990_) based on the following equation: ln (S*b*) = ln (S0) − *b*ADC. The ADC values were measured with W-L ROIs that were saved as described above for the same patient.

### Statistical analysis

Normality testing was performed using the Shapiro-Wilk test, and homogeneity of variance was evaluated using one-way ANOVA (Levene test). The numerical continuous variables are expressed as the mean ± standard deviation. The intraclass correlation coefficient (ICC) was calculated to evaluate intra- and interobserver variability. Interobserver agreement for W-L ROI and S-S ROI measurements was analysed by calculating the ICC and was interpreted as follows: 0.00–0.20, poor correlation; 0.21–0.40, fair correlation; 0.41–0.60, moderate correlation; 0.61–0.80, good correlation; and 0.81–1.00, excellent correlation. We also analysed the level of agreement (both inter- and intraobserver) by plotting the differences between the two measurements against the averages of the two measurements according to the method described by Bland and Altman (i.e., Bland-Altman plots). Differences between the two methods (W-L ROIs and S-S ROIs) were compared by paired-sample *t*-tests. The mean values of all parameters in the malignant group and the benign group were compared by independent-sample *t*-tests. Receiver operating characteristic (ROC) curves were generated to evaluate the diagnostic performance of IVIM and ADC parameters for distinguishing benign from malignant thyroid nodules. The optimal threshold was selected according to the Youden index. The area under the ROC curve (AUC) was calculated, and the sensitivity, specificity, positive predictive value (PPV), negative predictive value (NPV) and accuracy rate were determined. In addition, the AUC values of different parameters were compared with the *Z*-test proposed by DeLong et al. [26]. The parameters found to be statistically significant in the univariate analysis were subsequently entered into the multiple logistic regression models to determine their independent predictive value in predicting thyroid nodules. All statistical analyses were performed using MedCalc Online, version 16.2 (Medcalc Software, Mariakerke, Belgium) and SPSS (18.0 for Windows, SPSS, Chicago, IL). The adjusted *P* value was used for multiple comparisons. For all other statistical tests, a *P* value < 0.05 was used to indicate a statistically significant difference.

## Results

The results of normality testing showed that all parameters (i.e., *D*, *f* and D* derived from W-L ROIs and S-S ROIs) had approximately normal distributions, and Levene’s test revealed that all the variances were homogeneous (Table 1). Paired-sample *t*-tests of IVIM parameters measured using W-L ROIs and S-S ROIs demonstrated no statistically significant differences. The results of the intra- and interobserver reproducibility of the W-L ROI and S-S ROI analyses are provided in Table 2. The ICC values of all IVIM parameters (*D*, *f* and D*) using W-L ROI delineation were higher than those using S-S ROI delineation, and excellent intra- and interobserver reproducibility was obtained (range, 0.902–0.986 and 0.894–0.970, respectively). When an S-S ROI was applied, the intra- and interobserver reproducibility of the *D* value alone was excellent (ICC = 0.908 and 0.920, respectively), but the intra- and interobserver reproducibility for the D* value was only fair (ICC = 0.510 and 0.562, respectively). For the *f* value, the intraobserver agreement was moderate (ICC = 0.701), while the interobserver agreement was excellent (ICC = 0.846). The Bland-Altman plots for the IVIM parameters derived from W-L ROIs revealed small absolute intra- and interobserver variability, and the 95% limits of agreement (LoAs) for the IVIM parameters are presented in Table 3.

**Table 1:** Distribution of IVIM parameters derived from W-L ROI and an S-S ROI

	Reader 1 (*n* = 46)	Reader 2 first session (*n* = 46)	Reader 2 s session (*n* = 46)	Total (*n* = 138)	F	*P*^b^
*D*_*(W-L)*_	0.981 ± 0.375	0.973 ± 0.382	0.978 ± 0.366	0.977 ± 0.371	0.092	0.912
*f*_*(W-L)*_	0.352 ± 0.089	0.339 ± 0.093	0.332 ± 0.088	0.341 ± 0.090	0.077	0.926
*D*^a^_*(W-L)*_	11.178 ± 2.098	11.231 ± 2.242	11.193 ± 1.983	11.201 ± 2.095	0.168	0.845
*D*_*(S-S)*_	1.106 ± 0.525	1.005 ± 0.430	0.989 ± 0.446	1.034 ± 0.468	1.395	0.252
*f*_*(S-S)*_	0.295 ± 0.126	0.299 ± 0.144	0.319 ± 0.111	0.304 ± 0.127	1.502	0.226
*D*^a^_*(S-S)*_	11.126 ± 4.212	11.060 ± 4.069	10.507 ± 4.230	10.898 ± 4.150	0.158	0.854

W-L ROI (whole-lesion region of interest), S-S ROI (single-section region of interest), *D* (× 10^− 3^ mm^2^/s), *D*^a^ (× 10^− 3^ mm^2^/s)

Data are presented as the mean ± standard deviation. ^b^One-way ANOVA. F represents the statistical value

**Table 2:** Intra- and interobserver reproducibility of measurements of thyroid nodule IVIM parameters with different ROI methods

Parameters	W-L ROI ICC (95% CI)		S-S ROI ICC (95% CI)	
	Intraobserver	Interobserver	Intraobserver	Interobserver
*D*	0.986 (0.975–0.992)	0.970 (0.946–0.984)	0.908 (0.834–0.949)	0.920 (0.855–0.956)
*f*	0.929 (0.873–0.961)	0.951 (0.911–0.973)	0.701 (0.460–0.835)	0.846 (0.722–0.915)
D^*^	0.902 (0.823–0.946)	0.894 (0.808–0.941)	0.510 (0.114–0.729)	0.562 (0.208–0.758)

*D* (× 10^− 3^ mm^2^/s), D* (× 10^− 3^ mm^2^/s)

**Table 3:** Intra- and interobserver variation in the assessment of IVIM parameters with two ROI methods

	*D* (× 10^−3^ mm^2^/s)		D* (× 10^− 3^ mm^2^/s)		*f*	
	W-L	S-S	W-L	S-S	W-L	S-S
Intraobserver	−0.15-0.16	−0.48-0.51	− 0.09-0.07	−0.26-0.22	− 2.50-2.40	− 9.10-10.20
Interobserver	− 0.26-0.26	−0.60-0.40	− 0.10-0.10	−0.15-0.16	− 2.90-3.00	−8.30-8.20

Values are presented as the 95% limits of agreement (LoA; mean interdevice difference, spans of limits of agreement)

**Fig. 3:**
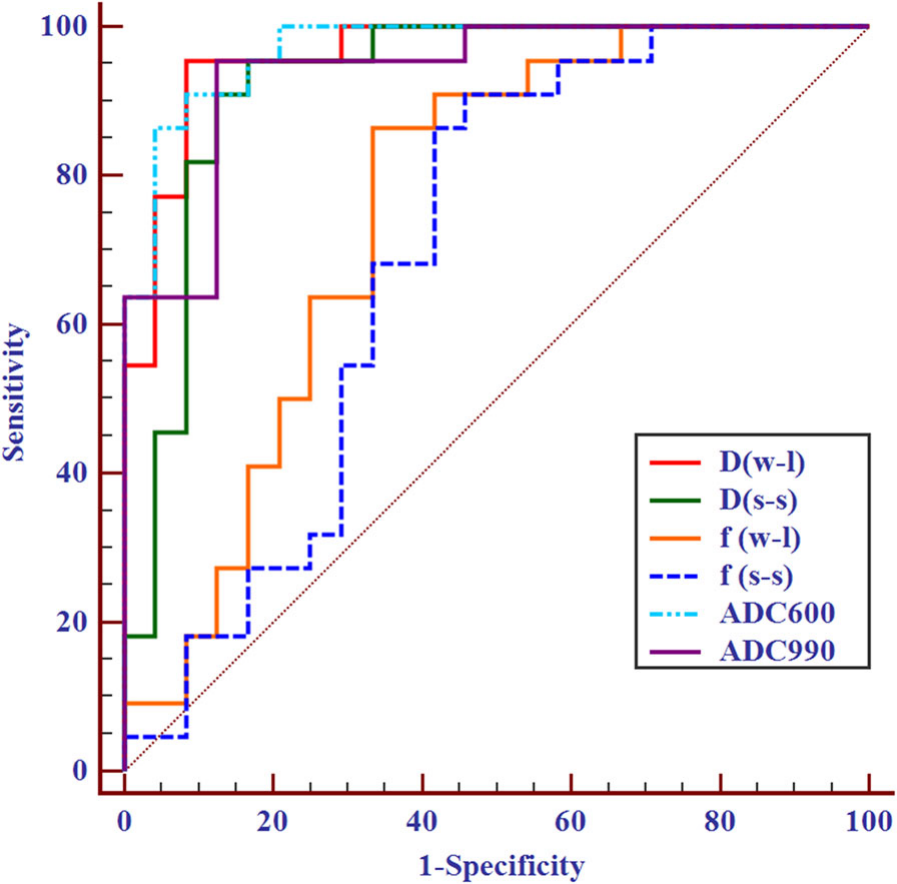
ROC curve analysis for discriminating malignant from benign thyroid nodules using IVIM and ADC parameters. W-L (whole-lesion ROI), S-S (single-section ROI)

**Fig. 4:**
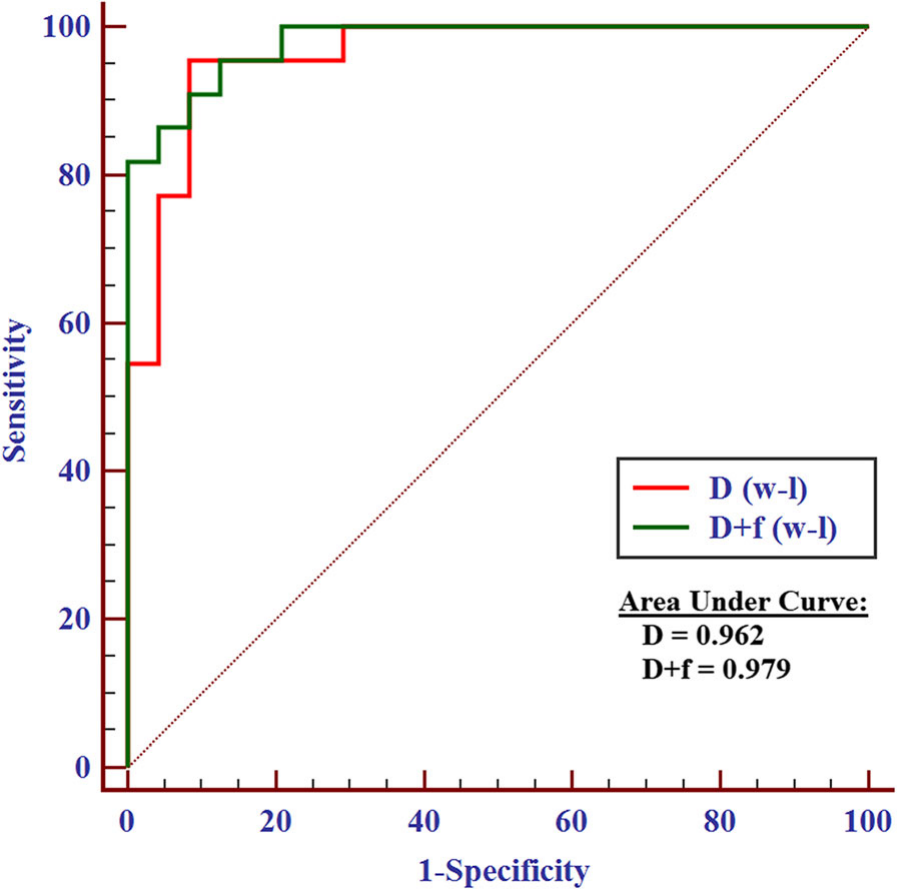
ROC curve analysis of D combined with f and D, respectively. W-L (whole-lesion ROI)

The results of independent-sample *t*-tests for benign and malignant nodules are summarized in Table 4. Among the three IVIM parameters derived from the two ROI methods, the *D* and *f* values were significantly lower in the malignant nodules than in the benign nodules. Their respective optimal cut-off values in the univariate and ROC curve analyses are summarized in Table 5 and are shown in Fig. 3. The cut-off values for *D* and *f* were 0.870 × 10^−^ 3 mm2/s (the *D* values of the benign nodules were greater than this value) and 35.30% (the *f* values of the benign nodules were greater than this value), respectively. The multivariate logistic regression showed that the AUC of *D* combined with the *f* value was not significantly higher than that of the *D* value [0.979 (95% confidence interval (CI), 0.886–1.000) vs. 0.962 (95% CI, 0.860–0.996), *P* = 0.461] (Fig. 4). The *D* and ADC_600_ values obtained from W-L ROIs achieved the highest AUC [0.962 (95% CI, 0.860–0.996) and 0.970 (95% CI, 0.871–0.998), *P* = 0.771] for differentiating malignant thyroid nodules from benign nodules.

**Table 4:** Comparison of IVIM parameters with two ROI delineations between benign and malignant groups

	Benign	Malignant	*P*
S-S ROI IVIM			
*D*	1.39 ± 0.47	0.70 ± 0.14	< 0.0001
*f*	35.93 ± 16.40	24.80 ± 7.06	0.0052
*D*^a^	11.08 ± 3.79	10.87 ± 3.03	0.8402
W-L ROI IVIM			
*D*	1.24 ± 0.35	0.69 ± 0.13	< 0 .0001
*f*	37.95 ± 10.05	29.36 ± 5.69	0.0010
*D*^a^	10.95 ± 2.55	11.54 ± 1.87	0.3782
W-L ROI ADC			
ADC_600_	2.08 ± 0.46	1.28 ± 0.15	< 0.0001
ADC_990_	1.71 ± 0.40	1.10 ± 0.17	< 0.0001

*D* (× 10^−3^ mm^2^/s), *f* (%), *D*^a^ (× 10^− 3^ mm^2^/s)

**Table 5:** Diagnostic performance of the IVIM and ADC parameters derived from the two ROI methods

	S-S ROI IVIM		W-L ROI IVIM		W-L ROI ADC	
	*D*	*f*	*D*	*f*	ADC_600_	ADC_990_
AUC	0.924	0.695	0.962	0.756	0.970	0.939
Youden index	0.788	0.451	0.871	0.530	0.826	0.830
Cut-off value	0.950	0.337	0.870	0.353	1.49	1.35
Sensitivity(%)	95.45	90.91	95.45	86.36	90.91	95.45
Specificity (%)	83.33	54.17	91.67	66.67	91.67	87.50
Accuracy(%)	89.13	71.74	97.83	76.09	89.13	91.30
PPV(%)	84.00	64.52	92.00	70.37	90.48	87.50
NPV(%)	95.24	86.67	95.65	84.21	88.00	95.45

*D* (× 10^−3^ mm^2^/s), ADC (× 10^− 3^ mm^2^/s)

## Discussion

The results reported in this study revealed that the IVIM parameters (*D*, *f* and D*) derived from W-L ROI delineation led to more reproducible and repeatable results than those derived from S-S ROI delineation. The diagnostic efficacy of the *D* value was almost as high as that of the ADC obtained from W-L ROI delineation. W-L ROI delineation of the thyroid to obtain IVIM measurements can be considered an effective method with excellent reproducibility for differentiating benign from malignant nodules.

In quantitative and qualitative analyses, unified delineation of lesions is very important. Most previous studies evaluating ADC values from thyroid nodules adopted S-S ROIs with solid areas, which did not include necrotic, cystic and micro-calcified areas [14–17]. However, most thyroid tumours are follicular-derived neoplasms, and the heterogeneity remained significant [2]. For this reason, S-S ROI delineation of solid areas may be associated with some problems. First, different observers or studies may select different slices for delineation. Second, even if the lesion is delineated on the same slice, the location and shape of the selected ROI are not always consistent. Third, the process may avoid visible cystic areas, but microcysts and micro-calcifications exist that cannot be avoided, leading to a decrease in the restrictive cell membranes and less hindrance to diffusion [2, 8]. The results derived from the S-S ROI analysis showed variability in both sensitivity and specificity [6, 7]. Whereas some studies have used W-L ADC imaging or histograms to evaluate thyroid nodules and distinguish different types of malignant thyroid nodules or benign nodules from malignant nodules [18–20], this study used IVIM with W-L ROI delineation that included cystic, necrotic and micro-calcified components of thyroid nodules, and the results demonstrated that all parameters had better reproducibility and repeatability than those of S-S ROI delineation. Notably, the perfusion-related parameters (*f* and D*) derived from the W-L ROIs showed excellent intra- and interobserver agreement, which may be related to the use of ROIs across whole tumours, thus minimizing the intra- and interobserver differences in ROI delineations. In addition, such differences may be a concern with the application of rFOV DWI, which employs a special 2D RF excitation pulse that is spatially selective in both the slice-select and phase-encoding directions. This method effectively provides high-resolution images with fewer artefacts and less distortion, providing more reliable and repeatable results to delineate the lesions more accurately [27, 28]. In our results, the intra- and interobserver agreement for the D* value was only fair in the S-S ROI analysis. Previous studies involving S-S ROI analysis of other organs have also shown that the repeatability of the D* or *f* values was poor [29–31], possibly because the analysis was associated with high uncertainty. A major challenge is the high variations in the signal-to-noise ratio (SNR) of the perfusion markers (D* and *f*), which has also been mentioned by several other studies [23, 32–34]. Furthermore, differences in the ROI analysis between the observers in this research who selected the slice of a lesion or delineation inconsistency may increase the uncertainty of D* values. The results of this study indicated that the repeatability of IVIM parameters is excellent when using W-L ROI analysis for thyroid nodules, providing a strong basic foundation for future multi-centre, large-sample studies as well as stratification research.

These results revealed that the diffusion effect plays a major role in differentiating malignant thyroid nodules, which may be based on differences in the cell density and histopathologic features of benign and malignant thyroid nodules [18, 35]. Some ADC and DKI studies with higher *b* values (1000 s/mm^2^ to 2000 s/mm^2^) have shown the role of diffusion in differentiating benign from malignant thyroid nodules [36, 37]. However, Tan et al. "s [11]. recently published study regarding IVIM in the thyroid showed that the *f* value achieved the highest AUC. The scanning parameter settings, signal receiving coil, delineation method, and proportions of pathological types of thyroid nodules used in their study were different from those in this study. Notably, selecting only the solid part of a lesion, i.e., the area where the microvasculature is likely to be densely distributed, may have yielded the perfusion effect reflected in the results, which was more obvious in our study than in their study.

Based on the excellent reproducibility of W-L ROI IVIM measurements, the ADC_600_ and ADC_990_ values of W-L ROIs were analysed. The results showed that the ADC values were higher than the *D* values, which may be related to the mix-up of perfusion factors in the bi-exponential model. This finding is consistent with those of other studies on the head and neck [8, 38]. IVIM was used to identify the effects of diffusion and perfusion (pseudo-diffusion). The *D* value was almost as good as the diagnostic efficiency because the ADC values were obtained from W-L ROIs. These results suggested that the IVIM model is an effective method for differentiating thyroid nodules. The AUC of *D* combined with the *f* value did not significantly differ from that of *D*. We interpreted the reasons for the above results as follows: 1) It may be related to different proportions of pathological types of thyroid nodules. Most lesions in our study were nodular goitres (22/46) and PTC (20/46), which showed higher percentages of cystic lesions and necrosis, thus reducing the overall vascularity and, consequently, confounding the results. 2) Expanding the sample size may render the results more realistic in reflecting the capillary blood flow and diffusion of water molecules. According to the fitting curve of the mathematical model, higher *b* values were believed to better approximate the true diffusion in the tissue. Wang et al. [39] reported that using the ADC value with the highest *b* value (2000 s/mm^2^) to differentiate benign from malignant tumours of the thyroid yielded a sensitivity of 96.15% and a specificity of 85.48%, indicating that a DWI sequence with a long acquisition time (14 min) may not be desirable for widespread use. In our research, the sensitivity and specificity of the *D* value in the W-L ROIs were 95.45 and 91.67%, respectively; the DWI sequence required approximately 5 min. Further exploration of the optimal *b* value that balances the SNR with scanning time and obtains high-quality DWI would be meaningful. On the other hand, recent studies using diffusion tensor imaging and other advanced diffusion imaging modules of the head and neck provide a reference for future thyroid research [40, 41].

However, the present study has several limitations. First, the sample size was relatively small, as the study was confined to a single centre, and the patients did not have a large variety of nodules, resulting in selection bias. The next step will be to estimate the required sample size for greater statistical power and collect data from multiple centres and large samples. Second, the S-S ROI approach used in this study is based on the delineation method used by most reports in the literature. However, this delineation does not include necrosis, cystic or microcalcification areas, which may have led to some missing information regarding quantitative parameters. Third, it would be useful to assess MRI performance in relation to thyroid imaging reporting and data system based on the ultrasound or histopathologic characteristics of thyroid nodules. Finally, these data were derived using a specific vendor sequence; other major vendors have offered similar sequences that can be applied to thyroid examinations, but further investigation is needed in a clinical setting.

## Conclusions

In conclusion, W-L ROI delineation to derive IVIM measurements with 2D RF rFOV DWI was confirmed to be a better method with excellent reproducibility and repeatability for differentiating benign from malignant thyroid nodules.

## Data Availability

All relevant data are available from the corresponding author.

## Abbreviations

ADC: Apparent diffusion coefficient
AUC: Area under the ROC curve
*D*: Pure molecular diffusion
*D**: Perfusion-related diffusion
DWI: Diffusion-weighted imaging
*f*: Perfusion fraction
FOV: Field of view
ICC: Intraclass correlation coefficient
IVIM: Intravoxel incoherent motion
LoA: Limits of agreement
NPV: Negative predictive value
PPV: Positive predictive value
RF: Radiofrequency
ROC: Receiver operating characteristic
ROI: Region of interest
S-S: Single-section
T1WI: T1-weighted imaging
T2WI: T2-weighted imaging
W-L: Whole-lesion
